# Treatment of Croup

**Published:** 1871-07

**Authors:** 

**Affiliations:** New York


### TREATMENT OF CROUP.

BY DR. BARKER, OF NEW YORK.

I always commence the treatment by an emetic of turpeth mineral, in doses of from three to five grains, according to the age of the child. If it does not act in fifteen minutes, I direct a second powder to be given. I regard it as very important that this emetic should be given immediately on the appearance of the symptoms which threaten croup. It is the only medicine which I have constantly carried in my pocket for twenty-eight years. In all families with young children that I attend, where the slightest tendency to catarrhal laryngitis has been manifested, I have been in the habit of directing that this medicine should be constantly kept where it can be readily found; and I have no doubt that at this moment a hundred families in this city have three-grain powders of the turpeth mineral carefully labelled "croup powders." There is one advantage in their use which I must not omit: if the supposed attack of croup is simply one of laryngismus stridulus, or of what is called false or spasmodic croup, the powders do no harm. If I find evidence of catarrhal laryngitis simply, then I rely mainly on opiates, which I regard as almost the specific for acute catarrh of the respiratory apparatus, whether it occurs in infantile or adult life. I direct full doses, proportionate to the age of the child, of Tully's powder or the Dovers powder, or the "Brown Mixture" of the United States Dispensatory. But I watch such a child closely, visiting it a second time bofore evening. But if, on my morning visit, I find the child with a quick pulse, hot skin, somewhat hurried breathing, and an occasional ringing cough, but with no thoraic rales, I direct that he shall be kept quiet in bed, comfortably covered, but not with too many clothes, and I prescribe the veratrum viride, in one or two drop doses, according to the age of the child, as for example in the following formula:

℞—Syr. simplicis, . . . ℥ i.

Aq. puræ, . . . ʒ i.

Spts. ether, nitrous . . scr. j.

Tinc. verat. viride, . . gtts. xvi–xxx. M

S. A teaspoonful every second hour.

I visit the child at least as often as every eight hours, and increase or diminish the dose according to the effect of the medicine on the pulse. I never am satisfied until I find the pulse below 80 per minute, and I then continue the veratrum in half the dose that I found necessary to bring it down to that point. My experience in the use of the veratrum viride now dates back more than twenty-five years, and I have never found it fail to reduce the pulse of irritation or of inflammation (it will not reduce the rapid pulse of exhaustion,) and I have never found the slightest danger or uncertainty in its use, as I watch its effects closely. If thoracic rales, hurried and labored respiration and other symptoms, indicate that the disease is travelling downward, I then substitute for the above prescription something like the following formula, of course varied according to the specific indications of the case:

℞—Mist acaciæ,

Syrup tolutanti, . . . aa. ℥ i.

Ammoniæ carb. . . . ʒ ss.

Tinc. verat. viride, . . . gtts. xvi—xxx. M

It has sometimes occurred that I have found evidence of increasing laryngeal and tracheal obstruction, and I have, in consequence, repeated the emetic of the turpeth mineral on the second or third day; but I have never had occasion or deemed it well to repeat it a third time.

In some cases of croup, in the advanced stages, when the respiration is hurried and irregular, the paroxysms of cough becoming less marked, the intermissions less distinct, the cough husky instead of ringorous, I have substituted for the last formula the following:

℞—Mist acaciæ
Syr. senegæ, . . . . aa. ℥ i.
Quiniæ,
Ammoniæ, . . . . aa. ʒ ss. M

S. To be well shaken. A teaspoonful every fourth hour.

When the croup is complicated with lobular pneumonia, I usually give the quinine separately, four or five grains, three times a day, while the little patient takes the last of the prescriptions containing veratrum viride.

I may be permitted to say that my great confidence in the treatment that I have sketched is based on the success which I have had. You, who know something of the extent and character of my practice, must admit that I have either been very lucky in never having had a case of true croup, or that I have had an unusual success in treating it; for, during the twenty years that I have practiced in this city, I have never lost a case from croup.

